# 2-Meth­oxy-4,6-di­phenyl­nicotino­nitrile

**DOI:** 10.1107/S1600536814001755

**Published:** 2014-01-31

**Authors:** Joel T. Mague, Alaa A.-M. Abdel-Aziz, Adel S. El-Azab, Ibrahim A. Al-Swaidan

**Affiliations:** a Department of Chemistry, Tulane University, New Orleans, LA 70118, USA; b Department of Pharmaceutical Chemistry, College of Pharmacy, King Saud University, Riyadh 11451, Saudi Arabia; c Department of Medicinal Chemistry, Faculty of Pharmacy, University of Mansoura, Mansoura 35516, Egypt

## Abstract

In the title compound, C_19_H_14_N_2_O, the phenyl rings form dihedral angles of 10.90 (10) and 42.14 (6)° with pyridine ring and an angle of 35.7 (2)° with each other. The orientation of the meth­oxy group is defined by the C—O—C—N torsion angle of 4.9 (2)°.

## Related literature   

For synthesis and drug-discovery studies of pyridine derivatives, see: Abdel-Aziz (2007 ▶); Abdel-Aziz *et al.* (2005 ▶); Cook *et al.* (2004 ▶); Upton *et al.* (2000 ▶); Al-Arab (1989 ▶); Perez-Medina *et al.* (1947 ▶). For related structures, see: Alvarez-Larena *et al.* (1994 ▶); Cao *et al.* (2009 ▶); Lv & Huang (2008 ▶); Mohamed *et al.* (2012 ▶); Patel *et al.* (2002 ▶).
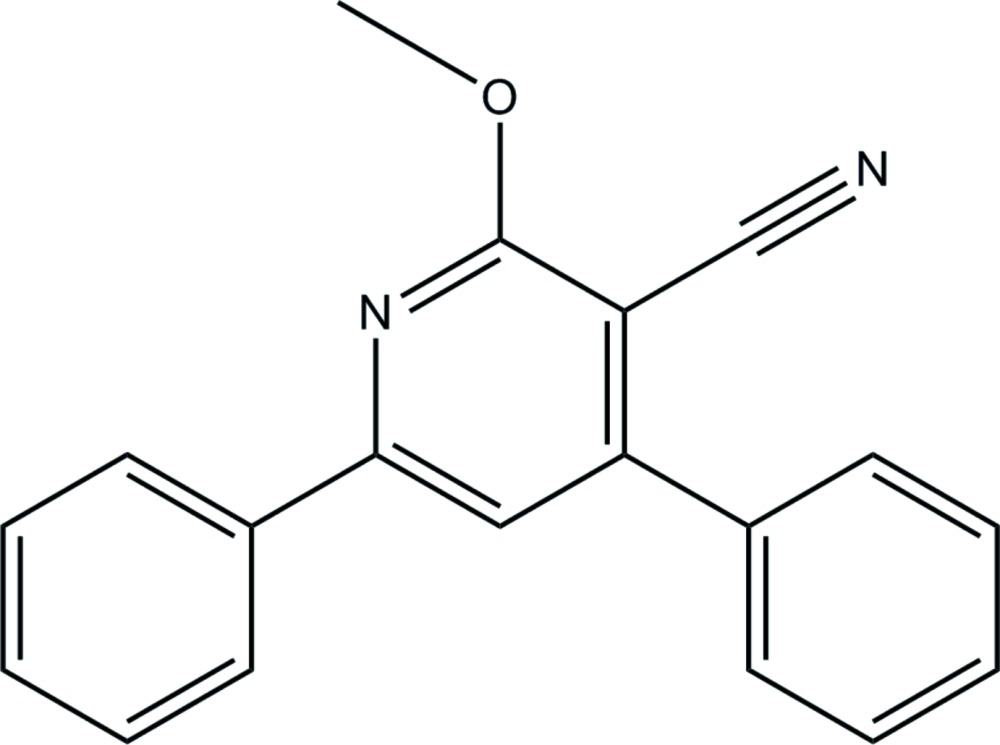


## Experimental   

### 

#### Crystal data  

C_19_H_14_N_2_O
*M* *_r_* = 286.32
Orthorhombic, 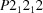

*a* = 15.0686 (16) Å
*b* = 24.327 (3) Å
*c* = 3.8986 (4) Å
*V* = 1429.1 (3) Å^3^
*Z* = 4
Mo *K*α radiation
μ = 0.08 mm^−1^
*T* = 100 K
0.22 × 0.11 × 0.06 mm

#### Data collection  

Bruker SMART APEXII CCD diffractometer
Absorption correction: multi-scan (*SADABS*; Sheldrick, 2009 ▶) *T*_min_ = 0.982, *T*_max_ = 0.995
12356 measured reflections
3344 independent reflections
2983 reflections with *I* > 2σ(*I*)
*R*_int_ = 0.047

#### Refinement  

*R*[*F* ^2^ > 2σ(*F* ^2^)] = 0.040
*wR*(*F* ^2^) = 0.093
*S* = 1.04
3344 reflections
200 parameters
H-atom parameters constrained
Δρ_max_ = 0.20 e Å^−3^
Δρ_min_ = −0.21 e Å^−3^

### 

Data collection: *APEX2* (Bruker, 2010 ▶); cell refinement: *SAINT* (Bruker, 2009 ▶); data reduction: *SAINT*; program(s) used to solve structure: *SHELXS97* (Sheldrick, 2008 ▶); program(s) used to refine structure: *SHELXL97* (Sheldrick, 2008 ▶); molecular graphics: *DIAMOND* (Brandenburg & Putz, 2012 ▶); software used to prepare material for publication: *SHELXTL* (Sheldrick, 2008 ▶).

## Supplementary Material

Crystal structure: contains datablock(s) I, global. DOI: 10.1107/S1600536814001755/lh5683sup1.cif

Structure factors: contains datablock(s) I. DOI: 10.1107/S1600536814001755/lh5683Isup2.hkl

Supporting information file. DOI: 10.1107/S1600536814001755/lh5683Isup3.cml

CCDC reference:

Additional supporting information:  crystallographic information; 3D view; checkCIF report

## Figures and Tables

**Fig. 1.**
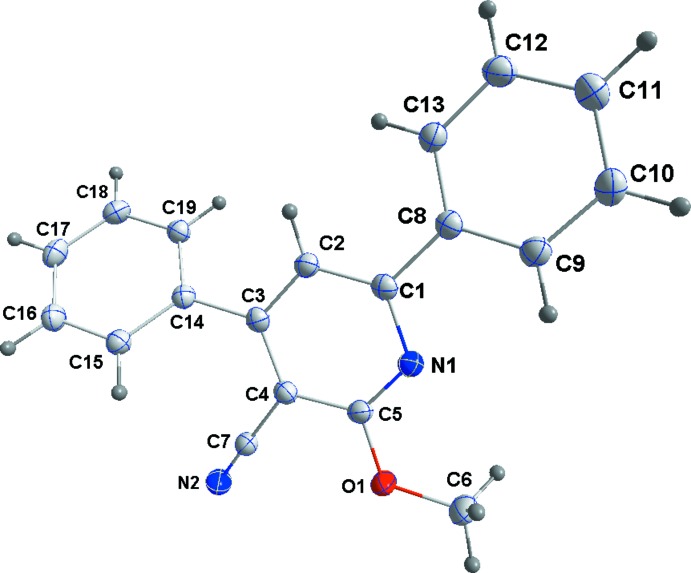
The molecular structure of the title compound. Displacement ellipsoids are drawn at the 50% probability level.

## supplementary crystallographic information

### 1. Comment

Compounds containing the pyridine nucleus are known to exhibit a large number of important biological properties (Perez-Medina *et al.*, 1947; Upton *et al.*, 2000; Cook *et al.*, 2004). As part of our ongoing program of drug discovery and design we report the structure of the title compound. The molecular structure of the title compound is shown in Fig. 1. The pendant phenyl rings (C8-C13) and (C14-C19) form dihedral angles of 10.90 (10) and 42.14 (6)°, respectively, with the central pyridine ring and a dihedral angle of 35.7 (2)° with each other. There are no significant intermolecular interactions which contrasts with the structure of the ethoxy analog where C—H···π and π—π stacking interactions are proposed (Patel *et al.*, 2002). The methoxy group is almost coplanar with the pyridine ring as indicated by the C6—O1—C5—N1 torsion angle of 4.9 (2)°. The conformations of related 2,4-diphenylpyridine derivatives differ from that of the title compound in the orientations of the pendant phenyl groups relative to the pyridine ring (Alvarez-Larena, *et al.*, 1994; Cao *et al.*, 2009; Lv & Huang, 2008; Mohamed *et al.*, 2012; Patel *et al.*, 2002).

### 2. Experimental

The title compound was prepared by the literature method (Al-Arab, 1989) and crystallized from acetone as slender, colourless plates.

### 3. Refinement

H-atoms were placed in calculated positions (C—H = 0.95 - 0.98 Å) and included as riding contributions with isotropic displacement parameters 1.2–1.5 times those of the attached carbon atoms.

### Crystal data

**Table:**

C_19_H_14_N_2_O	*F*(000) = 600
*M**_r_* = 286.32	*D*_x_ = 1.331 Mg m^−^^3^
Orthorhombic, *P*2_1_2_1_2	Mo *K*α radiation, λ = 0.71073 Å
Hall symbol: P 2 2ab	Cell parameters from 7963 reflections
*a* = 15.0686 (16) Å	θ = 2.7–28.4°
*b* = 24.327 (3) Å	µ = 0.08 mm^−^^1^
*c* = 3.8986 (4) Å	*T* = 100 K
*V* = 1429.1 (3) Å^3^	Plate, colourless
*Z* = 4	0.22 × 0.11 × 0.06 mm

### Data collection

**Table:**

Bruker SMART APEXII CCD diffractometer	3344 independent reflections
Radiation source: fine-focus sealed tube	2983 reflections with *I* > 2σ(*I*)
Graphite monochromator	*R*_int_ = 0.047
φ and ω scans	θ_max_ = 28.5°, θ_min_ = 1.6°
Absorption correction: multi-scan (*SADABS*; Sheldrick, 2009)	*h* = −19→20
*T*_min_ = 0.982, *T*_max_ = 0.995	*k* = −31→31
12356 measured reflections	*l* = −5→5

### Refinement

**Table:**

Refinement on *F*^2^	Primary atom site location: structure-invariant direct methods
Least-squares matrix: full	Secondary atom site location: difference Fourier map
*R*[*F*^2^ > 2σ(*F*^2^)] = 0.040	Hydrogen site location: inferred from neighbouring sites
*wR*(*F*^2^) = 0.093	H-atom parameters constrained
*S* = 1.04	*w* = 1/[σ^2^(*F*_o_^2^) + (0.0325*P*)^2^ + 0.477*P*] where *P* = (*F*_o_^2^ + 2*F*_c_^2^)/3
3344 reflections	(Δ/σ)_max_ < 0.001
200 parameters	Δρ_max_ = 0.20 e Å^−^^3^
0 restraints	Δρ_min_ = −0.21 e Å^−^^3^

### Special details

**Table:**

Experimental. The diffraction data were collected in three sets of 606 frames (0.3° width in ω) at φ = 0, 120 and 240°. A scan time of 40 sec/frame was used.
Geometry. All e.s.d.'s (except the e.s.d. in the dihedral angle between two l.s. planes) are estimated using the full covariance matrix. The cell e.s.d.'s are taken into account individually in the estimation of e.s.d.'s in distances, angles and torsion angles; correlations between e.s.d.'s in cell parameters are only used when they are defined by crystal symmetry. An approximate (isotropic) treatment of cell e.s.d.'s is used for estimating e.s.d.'s involving l.s. planes.
Refinement. Refinement of *F*^2^ against ALL reflections. The weighted *R*-factor *wR* and goodness of fit *S* are based on *F*^2^, conventional *R*-factors *R* are based on *F*, with *F* set to zero for negative *F*^2^. The threshold expression of *F*^2^ > σ(*F*^2^) is used only for calculating *R*-factors(gt) *etc*. and is not relevant to the choice of reflections for refinement. *R*-factors based on *F*^2^ are statistically about twice as large as those based on *F*, and *R*- factors based on ALL data will be even larger. H-atoms were placed in calculated positions (C—H = 0.95 - 0.98 Å) and included as riding contributions with isotropic displacement parameters 1.2 - 1.5 times those of the attached carbon atoms. 1280 Friedel pairs were left unmerged but the absolute structure could not be reliably determined.

### Fractional atomic coordinates and isotropic or equivalent isotropic displacement parameters (Å^2^)

**Table:**

	*x*	*y*	*z*	*U*_iso_*/*U*_eq_	
O1	0.43017 (8)	0.12053 (5)	0.0589 (4)	0.0260 (3)	
N1	0.57518 (9)	0.11317 (5)	0.2415 (4)	0.0213 (3)	
N2	0.38885 (10)	0.24495 (6)	−0.3038 (4)	0.0283 (3)	
C1	0.65658 (10)	0.13660 (6)	0.2753 (4)	0.0189 (3)	
C2	0.67269 (11)	0.19050 (6)	0.1762 (4)	0.0194 (3)	
H2	0.7302	0.2058	0.2047	0.023*	
C3	0.60488 (11)	0.22245 (7)	0.0350 (4)	0.0189 (3)	
C4	0.52180 (11)	0.19767 (7)	−0.0084 (5)	0.0198 (4)	
C5	0.51198 (11)	0.14286 (7)	0.1036 (5)	0.0208 (4)	
C6	0.41763 (12)	0.06623 (7)	0.1956 (6)	0.0310 (4)	
H6A	0.4557	0.0403	0.0720	0.046*	
H6B	0.3554	0.0554	0.1683	0.046*	
H6C	0.4332	0.0660	0.4396	0.046*	
C7	0.44794 (11)	0.22442 (7)	−0.1697 (5)	0.0215 (3)	
C8	0.72680 (11)	0.10115 (7)	0.4250 (4)	0.0194 (3)	
C9	0.70457 (11)	0.05036 (7)	0.5689 (5)	0.0226 (4)	
H9	0.6442	0.0392	0.5762	0.027*	
C10	0.76945 (11)	0.01607 (7)	0.7011 (5)	0.0250 (4)	
H10	0.7534	−0.0184	0.7976	0.030*	
C11	0.85806 (12)	0.03196 (7)	0.6928 (5)	0.0256 (4)	
H11	0.9028	0.0085	0.7821	0.031*	
C12	0.88043 (12)	0.08248 (7)	0.5528 (5)	0.0263 (4)	
H12	0.9409	0.0936	0.5473	0.032*	
C13	0.81601 (11)	0.11685 (7)	0.4214 (5)	0.0234 (4)	
H13	0.8324	0.1514	0.3277	0.028*	
C14	0.62283 (11)	0.28047 (7)	−0.0627 (4)	0.0190 (3)	
C15	0.56225 (11)	0.32239 (7)	0.0117 (4)	0.0218 (4)	
H15	0.5077	0.3140	0.1223	0.026*	
C16	0.58217 (11)	0.37627 (7)	−0.0770 (5)	0.0237 (4)	
H16	0.5413	0.4048	−0.0245	0.028*	
C17	0.66093 (12)	0.38876 (7)	−0.2411 (5)	0.0257 (4)	
H17	0.6737	0.4257	−0.3029	0.031*	
C18	0.72141 (11)	0.34755 (7)	−0.3158 (5)	0.0241 (4)	
H18	0.7753	0.3561	−0.4305	0.029*	
C19	0.70285 (11)	0.29378 (7)	−0.2222 (4)	0.0213 (3)	
H19	0.7451	0.2657	−0.2672	0.026*	

### Atomic displacement parameters (Å^2^)

**Table:**

	*U*^11^	*U*^22^	*U*^33^	*U*^12^	*U*^13^	*U*^23^
O1	0.0191 (6)	0.0201 (6)	0.0388 (7)	−0.0036 (5)	−0.0033 (6)	0.0000 (5)
N1	0.0203 (7)	0.0194 (6)	0.0241 (8)	−0.0007 (5)	0.0008 (6)	−0.0018 (6)
N2	0.0236 (7)	0.0269 (7)	0.0345 (8)	0.0006 (6)	−0.0047 (7)	−0.0002 (7)
C1	0.0193 (7)	0.0201 (7)	0.0172 (8)	0.0011 (6)	0.0013 (6)	−0.0032 (6)
C2	0.0183 (7)	0.0201 (8)	0.0197 (8)	−0.0004 (6)	−0.0003 (7)	−0.0032 (6)
C3	0.0208 (8)	0.0199 (7)	0.0160 (8)	0.0012 (6)	0.0020 (7)	−0.0024 (6)
C4	0.0184 (8)	0.0199 (8)	0.0210 (9)	0.0019 (6)	−0.0003 (7)	−0.0024 (7)
C5	0.0172 (8)	0.0213 (8)	0.0237 (9)	−0.0016 (6)	0.0018 (7)	−0.0037 (7)
C6	0.0254 (9)	0.0224 (9)	0.0451 (12)	−0.0059 (7)	−0.0031 (9)	0.0021 (8)
C7	0.0206 (8)	0.0198 (8)	0.0241 (9)	−0.0017 (6)	0.0017 (7)	−0.0013 (7)
C8	0.0207 (8)	0.0194 (8)	0.0182 (8)	0.0007 (6)	−0.0002 (7)	−0.0030 (6)
C9	0.0225 (8)	0.0214 (8)	0.0241 (9)	−0.0007 (7)	0.0020 (8)	−0.0010 (7)
C10	0.0296 (9)	0.0201 (8)	0.0254 (9)	0.0019 (7)	0.0021 (8)	0.0017 (7)
C11	0.0268 (9)	0.0252 (8)	0.0249 (9)	0.0056 (7)	−0.0005 (8)	0.0019 (7)
C12	0.0226 (8)	0.0286 (9)	0.0277 (9)	0.0005 (7)	−0.0037 (8)	−0.0010 (7)
C13	0.0249 (8)	0.0194 (8)	0.0259 (9)	−0.0015 (7)	−0.0012 (7)	0.0003 (7)
C14	0.0207 (8)	0.0191 (7)	0.0172 (7)	0.0004 (6)	−0.0031 (7)	−0.0009 (6)
C15	0.0209 (8)	0.0224 (8)	0.0222 (9)	−0.0006 (7)	−0.0005 (7)	−0.0016 (7)
C16	0.0264 (9)	0.0201 (8)	0.0245 (9)	0.0024 (7)	−0.0033 (7)	−0.0013 (7)
C17	0.0331 (9)	0.0202 (8)	0.0238 (9)	−0.0042 (7)	−0.0055 (8)	0.0020 (7)
C18	0.0233 (8)	0.0268 (9)	0.0222 (8)	−0.0045 (7)	−0.0009 (7)	0.0008 (7)
C19	0.0211 (8)	0.0225 (8)	0.0203 (8)	0.0009 (6)	−0.0013 (7)	−0.0032 (7)

### Geometric parameters (Å, º)

**Table:**

O1—C5	1.3583 (19)	C9—H9	0.9500
O1—C6	1.437 (2)	C10—C11	1.390 (2)
N1—C5	1.311 (2)	C10—H10	0.9500
N1—C1	1.359 (2)	C11—C12	1.386 (2)
N2—C7	1.147 (2)	C11—H11	0.9500
C1—C2	1.388 (2)	C12—C13	1.380 (2)
C1—C8	1.485 (2)	C12—H12	0.9500
C2—C3	1.397 (2)	C13—H13	0.9500
C2—H2	0.9500	C14—C19	1.395 (2)
C3—C4	1.400 (2)	C14—C15	1.399 (2)
C3—C14	1.487 (2)	C15—C16	1.388 (2)
C4—C5	1.411 (2)	C15—H15	0.9500
C4—C7	1.435 (2)	C16—C17	1.382 (2)
C6—H6A	0.9800	C16—H16	0.9500
C6—H6B	0.9800	C17—C18	1.386 (2)
C6—H6C	0.9800	C17—H17	0.9500
C8—C13	1.397 (2)	C18—C19	1.386 (2)
C8—C9	1.398 (2)	C18—H18	0.9500
C9—C10	1.385 (2)	C19—H19	0.9500
			
C5—O1—C6	116.07 (13)	C9—C10—C11	120.12 (16)
C5—N1—C1	117.68 (14)	C9—C10—H10	119.9
N1—C1—C2	121.80 (15)	C11—C10—H10	119.9
N1—C1—C8	115.97 (14)	C12—C11—C10	119.29 (16)
C2—C1—C8	122.23 (14)	C12—C11—H11	120.4
C1—C2—C3	120.49 (15)	C10—C11—H11	120.4
C1—C2—H2	119.8	C13—C12—C11	120.81 (17)
C3—C2—H2	119.8	C13—C12—H12	119.6
C2—C3—C4	117.52 (15)	C11—C12—H12	119.6
C2—C3—C14	119.74 (15)	C12—C13—C8	120.49 (16)
C4—C3—C14	122.74 (15)	C12—C13—H13	119.8
C3—C4—C5	117.62 (15)	C8—C13—H13	119.8
C3—C4—C7	123.46 (15)	C19—C14—C15	119.15 (15)
C5—C4—C7	118.89 (15)	C19—C14—C3	119.47 (14)
N1—C5—O1	119.45 (15)	C15—C14—C3	121.35 (15)
N1—C5—C4	124.86 (15)	C16—C15—C14	119.70 (16)
O1—C5—C4	115.69 (14)	C16—C15—H15	120.1
O1—C6—H6A	109.5	C14—C15—H15	120.1
O1—C6—H6B	109.5	C17—C16—C15	120.56 (16)
H6A—C6—H6B	109.5	C17—C16—H16	119.7
O1—C6—H6C	109.5	C15—C16—H16	119.7
H6A—C6—H6C	109.5	C16—C17—C18	120.19 (15)
H6B—C6—H6C	109.5	C16—C17—H17	119.9
N2—C7—C4	178.56 (19)	C18—C17—H17	119.9
C13—C8—C9	118.44 (15)	C17—C18—C19	119.64 (16)
C13—C8—C1	121.52 (15)	C17—C18—H18	120.2
C9—C8—C1	120.03 (14)	C19—C18—H18	120.2
C10—C9—C8	120.84 (16)	C18—C19—C14	120.72 (15)
C10—C9—H9	119.6	C18—C19—H19	119.6
C8—C9—H9	119.6	C14—C19—H19	119.6
			
C5—N1—C1—C2	1.6 (2)	C2—C1—C8—C9	170.16 (16)
C5—N1—C1—C8	−178.59 (15)	C13—C8—C9—C10	−0.8 (3)
N1—C1—C2—C3	−0.4 (2)	C1—C8—C9—C10	178.17 (16)
C8—C1—C2—C3	179.74 (15)	C8—C9—C10—C11	0.2 (3)
C1—C2—C3—C4	−1.4 (2)	C9—C10—C11—C12	0.3 (3)
C1—C2—C3—C14	178.68 (15)	C10—C11—C12—C13	−0.2 (3)
C2—C3—C4—C5	1.9 (2)	C11—C12—C13—C8	−0.4 (3)
C14—C3—C4—C5	−178.09 (15)	C9—C8—C13—C12	0.9 (3)
C2—C3—C4—C7	−175.93 (16)	C1—C8—C13—C12	−178.03 (16)
C14—C3—C4—C7	4.0 (3)	C2—C3—C14—C19	40.9 (2)
C1—N1—C5—O1	178.65 (15)	C4—C3—C14—C19	−139.07 (17)
C1—N1—C5—C4	−0.9 (3)	C2—C3—C14—C15	−137.09 (17)
C6—O1—C5—N1	4.9 (2)	C4—C3—C14—C15	42.9 (2)
C6—O1—C5—C4	−175.45 (16)	C19—C14—C15—C16	0.6 (2)
C3—C4—C5—N1	−0.9 (3)	C3—C14—C15—C16	178.63 (15)
C7—C4—C5—N1	177.11 (17)	C14—C15—C16—C17	0.6 (3)
C3—C4—C5—O1	179.55 (15)	C15—C16—C17—C18	−0.7 (3)
C7—C4—C5—O1	−2.5 (2)	C16—C17—C18—C19	−0.6 (3)
N1—C1—C8—C13	169.24 (16)	C17—C18—C19—C14	1.9 (3)
C2—C1—C8—C13	−10.9 (2)	C15—C14—C19—C18	−1.9 (2)
N1—C1—C8—C9	−9.7 (2)	C3—C14—C19—C18	−179.94 (16)
